# Future directions for chatbot research: an interdisciplinary research agenda

**DOI:** 10.1007/s00607-021-01016-7

**Published:** 2021-10-19

**Authors:** Asbjørn Følstad, Theo Araujo, Effie Lai-Chong Law, Petter Bae Brandtzaeg, Symeon Papadopoulos, Lea Reis, Marcos Baez, Guy Laban, Patrick McAllister, Carolin Ischen, Rebecca Wald, Fabio Catania, Raphael Meyer von Wolff, Sebastian Hobert, Ewa Luger

**Affiliations:** 1 SINTEF, Oslo, Norway; 2 University of Amsterdam, Amsterdam, The Netherlands; 3 Durham University, Durham, UK; 4 University of Oslo, Oslo, Norway; 5 CERTH, Thessaloníki, Greece; 6 University of Bamberg, Bamberg, Germany; 7 Claude Bernard University Lyon 1, Villeurbanne, France; 8 University of Glasgow, Glasgow, UK; 9 Ulster University, Jordanstown campus, Newtownabbey, UK; 10 Politecnico di Milano, Milan, Italy; 11 University of Goettingen, Göttingen, Germany; 12 University of Edinburgh, Edinburgh, UK

**Keywords:** Chatbots, Conversational agents, Dialogue systems, Future research directions, 68-02 Research exposition (monographs, survey articles) pertaining to computer science

## Abstract

Chatbots are increasingly becoming important gateways to digital services and information—taken up within domains such as customer service, health, education, and work support. However, there is only limited knowledge concerning the impact of chatbots at the individual, group, and societal level. Furthermore, a number of challenges remain to be resolved before the potential of chatbots can be fully realized. In response, chatbots have emerged as a substantial research area in recent years. To help advance knowledge in this emerging research area, we propose a research agenda in the form of future directions and challenges to be addressed by chatbot research. This proposal consolidates years of discussions at the CONVERSATIONS workshop series on chatbot research. Following a deliberative research analysis process among the workshop participants, we explore future directions within six topics of interest: (a) users and implications, (b) user experience and design, (c) frameworks and platforms, (d) chatbots for collaboration, (e) democratizing chatbots, and (f) ethics and privacy. For each of these topics, we provide a brief overview of the state of the art, discuss key research challenges, and suggest promising directions for future research. The six topics are detailed with a 5-year perspective in mind and are to be considered items of an interdisciplinary research agenda produced collaboratively by avid researchers in the field.

## Introduction

Chatbots are conversational agents providing access to information and services through interaction in everyday language. While research on conversational agents has been pursued for decades within fields such as social robotics, embodied conversational agents, and dialogue systems, it is only recently that conversational agents have become practical reality [77]. Key drivers of this development include advances in artificial intelligence (AI) fields, such as natural language processing (NLP) and natural language understanding (NLU), as well as the increased consumer uptake of platforms conductive to conversational interaction [38].

Chatbots are currently taken up in application areas as diverse as customer service [1], health [105], education [53], and office work [78]. There has lately been a marked increase of interest in chatbot research within academia and industry, specifically from 2016 and onwards [86]. Recent research addresses, for example, chatbot use (e.g. 74], interaction design (e.g. 57] and assessment (e.g. 63], as well as specific applications (e.g. 96] and technological advances (e.g. 2].

The rapidly growing body of chatbot research has a marked interdisciplinary character—spanning fields such as informatics, management and marketing, media and communication science, linguistics and philosophy, psychology and sociology, engineering, design, and human-computer interaction. This broad emerging knowledge base is valuable, but also implies that research of relevance to chatbots is currently fragmented across disciplines and application domains. With a broad and rich range of chatbot applications, it is imperative to understand why certain chatbot usages are working (or not) by referencing in-depth theoretical frameworks. As the current interdisciplinary wave of chatbot research is progressing, there is a need to define overarching research directions for guidance, allowing new studies and initiatives to systematically build on and benefit from existing work.

In this paper, we propose a research agenda which has been distilled through a series of dedicated workshops on chatbot research—CONVERSATIONS—with intensive discussions among researchers and practitioners actively working on chatbots. The research agenda has the overall aim to motivate and guide research to establish requisite knowledge for fully realizing the potential of chatbots as a powerful means of accessing information and services and for understanding the impact of chatbots at the individual, group, and societal level. As the research on chatbots is rapidly evolving, we hold that deriving a research agenda from collaborations and discussions among avid researchers and practitioners, who keep abreast of the ongoing developments of the area, is a more effective approach as compared, for example, with a mapping study or systematic literature review. Furthermore, this collaborative approach enables us to gain insights from different perspectives to address opportunities, challenges, and perceived research needs within the field. The research agenda serves as a concise research roadmap, offering links to pertinent studies for those readers who are interested in delving further into specific fields.

In the following, we first present relevant background on chatbot research before we detail the need for a consolidation of future directions. We then present our approach and proposed set of directions. Finally, we discuss our proposal and the way forward.

## Background

### Historical roots of chatbot research

The emerging chatbot research area has its historical roots in several research fields addressing different aspects of conversational computer systems—the most prominent of these with decades of research and efforts at industrial applications. Within the field of dialogue systems [77], researchers have since the sixties and seventies worked on text based [12] and later spoken [59] conversational user interface to support users with specific tasks. Other streams of research preceding and relevant to current chatbot research have addressed conversational interaction with physical social robots [15], and embodied virtual agents [18]. There has also been a long-term research initiative addressing computer systems for open-domain small talk [98], including the development of the artificial intelligence markup language [111] used to power chatbots for social chit-chat. Conversational computer systems have also had a long and, at times, winding path through various commercial applications—particularly automated solutions for customer service, sales and support [72], including interactive voice response (IVR) systems for phone-based self service [23].

The recent substantial increase in chatbot research can be seen as a direct response to the uptake of so-called virtual assistants by big tech companies, specifically the inclusion of Siri as part of Apple operating system in 2011, Amazon's promotion of Alexa since 2014 and the conversational turn of Facebook, Microsoft and Google in 2016 [25]. Piccolo et al. [86] concluded that chatbot research has followed in the trail of the industrial uptake of conversational computer systems rather than being at the driver seat. In consequence, the contribution of this burgeoning research area is as much to understand the emerging application, uses, and implications of conversational computing systems, as to improve on their technological underpinnings and methods for design and development. Consequently, the chatbot research area has a broader scope and disciplinary coverage than the fields at its historical roots.

### Clarification of terminology

As noted by McTear [77], research streams such as those of dialogue systems, embodied conversational agents, and social robotics, are now converging in a common aim for developing and improving on conversational user interfaces to computer systems. However, there is still a wide variety of terms in use in reference to the object of this converging research interest. Since the recent industrial uptake of conversational computing systems, these have increasingly been referred to as chatbots within industry and media [25] and also in research. To demarcate the research area driven by the industrial uptake of conversational computer systems, and to signify the attention of this area towards emerging patterns of use, as well as broader business and societal implications, we refer to this area as chatbot research.

In line with this scoping of the research area, we understand chatbots as *conversational agents providing access to information and services through interaction in everyday language*—an understanding which is in line with the definitions by Følstad and Brandtzaeg [39] and Hobert and Meyer von Wolff [54]. This use of the term chatbot encompasses conversational agents for goal-oriented task completion, informational purposes, entertainment, and social chatter. It also encompasses agents supporting interactions through text, voice, or both. The use of the term is in reference to the object of our research interest—current and future design, development, and implications of information and services provided through conversational computer systems—rather than in reference to a specific set of technologies or approaches.

In consequence, our use of the term chatbot is broader than what may be found in other research streams. For example, some distinguish between voice-based and text-based conversational agents, using the term chatbot to refer to the latter, (e.g. Ashktorab et al. 6). Others distinguish between conversational agents for goal completion versus social chatter, referring only to the latter as chatbots (e.g. Jurafsky and Martin 61). However, in consequence of the rapid evolvement both in technology, services, and patterns of use, we find such attempts at principled scoping of the chatbot term challenging. For example, there is often no clear distinction between social chatter and goal-orientation in conversational agents—as seen by the importance of social responses for customer service chatbots [114]. Likewise, the distinction between text and voice is less than clear-cut as the same conversational agents may make use of different modalities [97].

### Enablers of current chatbots

Current chatbots are enabled by a large range of technologies and services [97] at varied levels of sophistication. Dialogue management may be enabled through simple rule-based approaches, statistical data-driven systems, or neural generative end-to-end approaches [77], and many systems employ hybrid models [50]. Whereas early chatbots for social chit-chat may exemplify rule-based approaches (e.g., Weizenbaum 112) current statistical data-driven systems—such as chatbots for customer service—have user intents and corresponding chatbot responses identified on the basis of training of machine learning models based on example user data [66]. Generative chatbots based on end-to-end approaches are currently a research topic of substantial interest. A much-cited example is presented by Vinyals and Le [109]. More recently, Facebook's Blender [90] and Google’s Meena [2] have received substantial interest due to their near-human open domain conversational capabilities.

A large number of general-purpose platforms and frameworks are available for chatbot delivery, such as Google's DialogFlow,^1^ Microsoft Bot Framework,^2^ Pandorabots,^3^ and the open-source frameworks Rasa^4^ and Mycroft.^5^ The platforms range from so-called low-code alternatives [26], where implementation and maintenance may be conducted with limited or no software engineering skills, to frameworks serving as basis for larger software development projects. Platforms and frameworks for chatbot delivery typically provide integrations with a range of communication channels, including social media and chat, as well as websites and collaborative work support systems. Hence, the same chatbot may reach users across their preferred channels.

### Research communities

Chatbot research is currently evolving within and across a range of disciplines and has a strong interdisciplinary character. Ground-breaking research has been presented in fields as diverse as communication (e.g. Go and Sundar 42), health (e.g. Fitzpatrick et al. 35), informatics (e.g. Adiwardana et al. 2), and business (e.g. Adam et al. 1). While dedicated workshops and conferences of relevance to chatbot research are emerging—such as CUI,^6^ CONVERSATIONS,^7^ and CAIR^8^—in addition to established venues—such as SIGDIAL,^9^ IVA,^10^ IWSDS,^11^ and INTERSPEECH^12^—research findings are typically presented in a broad range of journals and conferences. Research related to chatbots is also conducted in multiple communities with varying degrees of exchange among them. These communities may not label their area of interest as chatbot research but, for example, research addressing *conversational agents* [79], *dialogue systems* [59] or *social robotics* [93]. The research objectives within these communities may only be partially overlapping. However, we believe these communities likely will benefit from strengthening their collaboration and mutually inform and support each other's research.

## Objective: to propose future research directions

While there is a rapidly expanding body of knowledge relevant to chatbot research, rooted in long-standing research fields, current research and knowledge are fragmented across disciplines, application areas, and communities. Such fragmentation is to be expected in a rapidly expanding field. However, we are now at a point in time where it is beneficial to stake out common directions for future research.

The identification of common research directions is not something that can be achieved by individual researchers or single communities. Rather, it should be seen as a collaborative and continuously evolving process across individuals and communities, where adjustments are made on the basis of new insights and knowledge as it is gathered.

Our objective in presenting this work is therefore to provide a needed interdisciplinary and collaborative basis to initiate and guide a broader discussion on the key future research directions for chatbot research. As such, the work will provide a broader perspective on research directions than what is provided, for example, in current reviews on chatbots within specific domains (e.g. [105, 78]), specific aspects of chatbot technology and design (e.g. [20, 86]), or user behaviour and experience (e.g. [63, 119]).

Furthermore, we address perspectives and topics for chatbot research which may be more broadly scoped than what may be found within, for example, the fields and disciplines in which chatbot research has its roots. As such, we aim for the work to provide a basis for chatbot research that is seem of value to research and practice alike, and which also may serve to bridge relevant research currently embedded in distinct disciplines.

## Approach

The proposed future research directions are based on the collaborative work conducted as part of the CONVERSATIONS workshops. CONVERSATIONS is an international workshop series for chatbot research, where researchers, students, and practitioners with interest in chatbots gather to present their work, discuss, and collaborate. The first workshop in this series was organised in 2017 and it has since been a yearly event, advancing from being arranged in conjunction with a research conference the two first years to now being a 2-day stand-alone event. The most recent workshop in 2020 [41], arranged as a virtual event due to the COVID-19 pandemic, involved about 150 registered participants from more than 30 countries and 80 different organizations, including more than 20 paper presentations. The participants represent disciplines such as computer science, information systems, human–computer interaction, communication studies, linguistics, psychology, marketing, and design.

Throughout the CONVERSATIONS workshops, we have discussed chatbot research challenges and how to address these. In the first CONVERSATIONS workshop (2017), approximately half of the overall 30 participants engaged in identifying and clustering key research challenges of the field into overarching research topics. The research challenges within these topics formed the basis for the call for papers to the later CONVERSATIONS workshops (2018, 2019, 2020). At the third CONVERSATIONS workshop (2019), the topics—updated throughout the workshop series—were revisited through in-depth group discussions involving approximately half of the overall 50 workshop participants. The output from these group discussions forms the basis for the presented research directions.

The deliberative process at the workshop series was key to identify and propose research directions in a true interdisciplinary fashion. In the 2019 edition, workshop participants were assigned to groups—each with the mandate to address one of six topics: (a) user and communication studies, (b) user experience and design, (c) frameworks and platforms, (d) chatbots for collaboration, (e) democratizing chatbots, and (f) ethics and privacy. The group work was conducted in two sessions across the 2 days of the workshop. In the first session, each group carefully discussed the research topic in a 5-year time frame, identifying (a) relevant state of the art, (b) key research challenges, and (c) future directions. In the second session, the output of each group was presented to the workshop plenary and discussed.

The collaborative process extended across the following year, taking into account the contributions and discussions of the CONVERSATIONS 2020 workshop as well. As a result, the proposed research directions reflect the interdisciplinary position of a group of collaborating researchers within this emerging field.

## Proposed future research directions

Through the CONVERSATIONS workshop series, six overarching topics for future chatbot research have been identified. In the following, we detail each of these based on the CONVERSATIONS output, with particular concern for the state of the art, research challenges, and future research directions. An overview of the six topics and associated future research directions is provided in Table 1.

**Table 1:** Topics and proposed directions for future research on chatbots

Topics	Proposed future research directions
1. Users and implications	(a) Emerging chatbot user groups and behaviours
	(b) Social implications of chatbots
2. Chatbot user experience and design	(a) Design for improving chatbot user experience
	(b) Modelling and evaluating chatbot user experience
3. Chatbot frameworks and platforms	(a) Interpretation capabilities and context understanding
	(b) Emerging techniques for chatbot design and testing
4. Chatbots for collaboration	(a) Modelling human-chatbot collaboration
	(b) Empirical investigations of human-chatbot collaboration
5. Democratizing chatbots—chatbots for all	(a) Chatbots for social good
	(b) Inclusive design with and for diverse user groups
6. Ethics and privacy in chatbots	(a) Understanding chatbot ethics and privacy
	(b) Ethics by design

### Users and implications

Given the current evolving use and emerging use cases for chatbots, important questions to ask concern chatbot users and their contexts of use. This includes investigating antecedents for chatbot use—namely individual characteristics, motivations and boundary conditions for choosing, accepting or even preferring to interact with conversational agents. Furthermore, it is necessary to explore and discuss implications of chatbot use on individuals, groups, organizations and society at large.

#### State of the art

Chatbot use is becoming commonplace. For example, in 2019, over 50 % of US and German consumers were estimated to have used chatbots at least once—with even higher numbers in the UK or France [88]. In consequence, chatbot researchers currently have an unprecedented opportunity for real-world study of users [106], user motivations [14], and implications at scale. In consequence, knowledge on chatbot use has been gathered for a range of contexts—in the private sphere [87], at work [74], and in public spaces [17].

A substantial body of research of relevance for chatbot use has been developed within broad domains such as health [105], education [84], and business [8], as well as more specific application domains such as polling [62], information search [73], libraries [92], and museums [64]. Knowledge of relevance for understanding the impact of chatbots on individual users may be found in studies of therapy chatbots (e.g. [35]), relational agents (e.g. [10] and chatbots for social relationships [103]. Specifically, it is of interest to note how such studies address implications of individual long-term use.

Because of this, we have substantial knowledge on potential and actual chatbot users and implications for individuals across a wide variety of contexts, building upon a rich stream of research dating back to the work of Weizenbaum [112]. Chatbot impact on society has, however, not been comprehensively researched and only tentatively been suggested in studies of chatbots for specific domains—as mentioned above. This may in part be due to the substantial impact on the level of organizations and society is assumed to be seen in the future more so than the present.

#### Research challenges

While we have substantial knowledge on current chatbot users, important topics lack sufficient coverage. Two warrant particular mention: (a) broader chatbot uses and user groups and (b) implications of chatbot use, both detailed below.

For the broader chatbot uses and user groups, the rich literature needs to be continuously updated, especially when it comes to user motivations and behaviour of emerging user groups. This includes knowledge on specific demographics, for example, vulnerable users, such as children, elderly and users with special needs, as well as user groups within particular application areas. Moreover, research still needs to assess whether there are systematic differences in the adoption and usage of chatbots driven by socio-demographic characteristics.

Implications of chatbot use entail a range of exciting research challenges, as knowledge is needed on how the uptake of chatbots may impact groups, organizations, businesses, and society at large. For example, as chatbots are taken up by different sectors and industries, chatbots may transform service provision and work processes.

Another example is our need for knowledge on how the interaction patterns that emerge between human users and chatbots may spill over to our interaction with other people: Will the demanding communication style we learn to use for virtual assistants, such as Alexa and Siri, impact our communication style with our partners or collaborators? How will the companionship offered by social chatbots influence users' social lives and desires, and how chatbots may enter the social fabric of groups or organizations?

#### Future research directions

Based on the current state of the art and identified research challenges, two future research directions emerge as particularly promising in the area of chatbot user and communication studies.

- *Emerging chatbot user groups and behaviours.* While there exists knowledge on current chatbot user groups, this needs to be updated as technology, services, and patterns of use evolve. Furthermore, there is a need to move from studies of chatbot users in general to studies of chatbot users and behaviours for particular demographics, domains, or contexts. We are beginning to see this for domains such as health, education, and business, but given the uptake of chatbots in new contexts and domains, this is an area of research which will be in continuous need of update.
- *Social implications of chatbots.* The study of social implications of chatbots is an area where we expect to see substantial research interest in the near future. Knowledge of the social implications of chatbot use will be of importance to guide also future development and design of chatbot services. Possibly, a string of research on the broader social implications could be motivated from the broader discourse on implications of AI for labour and business (e.g. [37, 76]). It will be beneficial to accommodate for research on unintended social consequences of chatbots or how chatbots are shaped in response to its uptake in society.

### Chatbot user experience and design

Chatbot user experience and design concerns how users perceive and respond to chatbots, and how chatbot layout, interaction mechanisms and conversational content may be designed so as to manage these perceptions and responses. To gather insight into users' perceptions and responses, and how these are impacted by chatbot design, user-centred evaluations of chatbots is necessary; that is, assessments of users' perceptions and responses to chatbots conducted through established methods.

#### State of the art

Chatbot user experience has been a key theme in recent research efforts, for voice-based virtual agents [75] and text-based applications [4]. This has helped identify factors contributing to positive or negative user experience [118] and has addressed specific aspects such as trust [119], perceived social support [71], human likeness [4], and how these aspects are impacted by chatbot design [42]. There is also a growing base of research to inform design of chatbot interactions, whether this concerns conversational design [6], personalization of chatbots [69], the use of interactive elements in chatbots [57], or the use of social cues to indicate social status and capabilities [32]. Recently, a number of textbooks (e.g. [48, 79, 97]) and industry guidelines (e.g. by Google^13^ and Amazon^14^) have also been published on chatbot interaction design and conversational design. Textual and acoustic properties of users' dialogue input are gradually being applied as outcomes in empirical research for studying engagement and experience with conversational agents [52, 67]. Furthermore, there exists an extensive body of research on emotion detection through speech (e.g. [95]) and non-verbal behaviour [27] of high relevance to chatbot user experience and design.

There is also a grown body of knowledge on methods and measures for evaluating chatbot user experience. User-centred evaluation has been key to research within several of the disciplines at the roots of current chatbot research, such as studies of social presence in social robotics [82] and the use of user satisfaction measures in evaluations of dialogue systems [28]. Evaluation in chatbot research is conducted by instruments for users' self reports of user experience [63], through user observation and interviews [75] and analyses of chatbot interaction [66], and also by physiological measurements [22]. A range of evaluation approaches are employed, including experiments by self-administered online studies [5] or in the lab [22], observational studies in the wild [64], and investigations of long-term interactions with established services [73].

#### Research challenges

While there is a growing body of research available on chatbot user experience there still is a lack of knowledge on how to leverage the findings from this research in chatbot designs that consistently delight and engage users. Users still experience issues in chatbot interaction, both in terms of pragmatic experiences—where chatbots fail to understand or to help users achieve their intended goals [75]—and in terms of hedonic experiences—where chatbots fail to engage users over time [117]. These issues may in part be seen as due to the more general challenge of designing human-AI interaction [116]. There are indeed indications that these challenges are being mitigated, for example in the case of improvements in customer service chatbots [80] and in the uptake of social chatbots such as Replika [103]. However, the strengthening of chatbot user experiences remains a key research challenge.

Related to the challenge of strengthening chatbot user experience, is the challenge of measuring and assessing chatbots in terms of user experience and from a more holistic perspective to determine whether chatbots are actually beneficial. Relevant aspects for this are, for instance, usefulness, efficiency and process support. While there is a large number of studies on chatbot user experience available, there is a lack of common definitions, metrics and validated scales for key aspects of chatbot evaluations [63]. Furthermore, while a broad range of approaches are employed there is a lack of commonly applied approaches to evaluation.

#### Future research directions

Future research should be directed at addressing the identified key research challenges. Specifically, the following two directions are proposed.

- *Design for improving chatbot user experience.* Future research on chatbot user experience needs to evolve from exploring and assessing aspects of user experience and effects of chatbot design elements, towards studying how this knowledge may impact and improve chatbot user experience in industrial applications. Specifically, to translate findings of theoretical interest to conclusions of practical impact on design. This is not to say that research to build theory on chatbot user experience is not needed, but this research may need to take up also more design-oriented objectives—so as to condense current research and knowledge to guidelines that may directly inform conversational design or interaction design.
- *Modelling and evaluating chatbot user experience.* To advance future research on chatbot user experience, there is also a need for convergence of chatbot user experience models, measurements, and approaches to evaluation. While diversity in definitions and operationalizations is to be expected in an emerging field of research interest, there may now be the time for seeking agreement and consistency in the use of terminology and definitions of user experience constructs, and also to identify and apply standardized measurements (benchmarks) for these constructs. While such convergence should not be done in a way that hampers theoretical advancement and method innovation, there is clearly a benefit in including common measurements across studies so as to enable cross-study comparison and aggregation, and to be able to track progress over time. For this purpose, established evaluation approaches from fields such as human-computer interaction or the tradition of dialogue systems may be beneficial.

### Chatbot frameworks and platforms

This area of chatbot research concerns the current and future frameworks and platforms for chatbot development and delivery. That is, the technological underpinnings of chatbot implementations such as solutions for natural language processing, data extraction, storage, and access, as well as mechanisms to identify and adapt chatbot interactions to context and user profile.

#### State of the art

The advances in chatbot frameworks and platforms are key enablers of the current interest in chatbot applications. As noted in the background Sect. 6 myriad platforms and frameworks are available to support design and development of chatbots. Key advances include the application of supervised machine learning for classification and information retrieval—enabling, for example, intent prediction and identification of user sentiment [20], which are critical to support task-oriented conversations. Furthermore, the use of generative approaches has seen substantial progress, where end-to-end dialogue systems are applied to predict suitable responses to user input based on models built from large conversational datasets [2, 109]. Finally, the introduction of the Transformer [107] as a dominant and highly effective architecture for natural language processing along with high-quality open-source libraries [113] have lowered the barrier to entry and make it possible to build conversational models that exhibit high generalization and coherence [90].

In this regard, large-scale generative models are becoming increasingly impactful, enabling a wide range of tasks that can benefit chatbot development [36]. Models such as GPT-3 [16] by OpenAI and BERT (Bidirectional Encoder Representations from Transformers) [29] by Google leverage massive amounts of data and computational power that would not be available to smaller players. Indeed, GTP-3 currently uses 175 billion parameters, and it is estimated to have cost 12 million US dollars to train [36]. Thus, opening up these powerful models to the public has the potential to accelerate chatbot development even further. It is important to note, however, that criticism around large models has been growing lately [9], especially ethical concerns regarding undesirable and often inscrutable societal biases percolating the models [9, 120], carbon footprint [9, 99], misuse and misinterpretation [9], privatization of AI research [99], and even research opportunity costs [49].

#### Research challenges

While substantial advances have been made in chatbot frameworks and platforms, a number of challenges remain. Specifically, we lack the needed technological underpinnings to support some key aspects of chatbot applications. We see four such challenges of particular importance. First, understanding user input remains difficult. While machine learning approaches have strengthened both natural language understanding and intent prediction, chatbot interaction is prone to conversational breakdowns due to interpretation issues—in particular in everyday situations or in the wild [87]. Second, the challenge of modelling and adapting to the user and conversational context is as important as ever. For example, as chatbots are being increasingly deployed in the health domain, in possibly sensitive scenarios, it becomes of paramount importance for chatbots to adapt the conversation to social, emotional and even the health literacy aspects of users [60]. These were identified as key challenges already by Weizenbaum [112] and have remained such ever since. Third, challenges remain in solutions for supporting chatbot development and standardised testing for example in terms of studies simulating production environments and approaches to improve chatbots more easily in production. Last, as chatbots are becoming part of an ecosystem of software systems, supporting chatbot integration in this context is a new emerging challenge—for example by facilitating conversational presentation of information and content also intended for other use [7].

#### Future research directions

- *Interpretation capabilities and context understanding.* As in recent years, further progress in the field of chatbots will depend on advances in natural language understanding, which will remain a key area of research interest. To enable progress in natural language understanding, more quality training data in open repositories is needed. Also, new techniques supporting the involvement of domain experts in content development, natural language processing, and dialogue management—through low-code or end-user development approaches—may be relevant. Finally, the challenges of context and user understanding, for sustained dialogue and adaptation of conversations, will remain critical aspects of future research.
- *Emerging techniques for chatbot design, development, and deployment.* Future research is needed to provide increases support for design, development and deployment. The deployment of conversational interfaces on top of software-enabled services is a promising direction for chatbot research and implementation (e.g. [115])—enabling digital assistants' access to information and services currently out of their reach, and rendering existing systems more accessible. In terms of design, it is promising to see that general guidelines for human-AI interaction are emerging [3] and more of these are needed. There is also a need for guidelines drawn from systematic comparative studies and to embed research-derived guidelines into chatbot frameworks.

### Chatbots for collaboration

The area of chatbots for collaboration concerns how we may understand and design chatbots in the context of networks that comprise humans and intelligent agents, for example for social networking, teamwork, or service provision. While the current research on chatbots typically addresses dyadic interactions between one chatbot and one user, we foresee that chatbots in collaborative relations involving more people and bots will become more prominent as chatbots mature further. In addition, we consider that collaborative relations can be addressed to a chatbot's relations with external online services in the form of application programming interfaces (APIs) and other artificial agents.

#### State of the art

Chatbots for collaboration concerns chatbots involved in interactions with humans and possibly with other chatbots in networks larger than dyads. While not as prominent as chatbots for simpler dyadic interaction, chatbots for collaboration have been developed and implemented in a range of contexts and for various purposes, for example, to support group processes in education [43], at work [11] and organizational settings [104], as well as in gaming communities [96].

Types of collaboration with chatbots may include (a) one human collaborating with one chatbot as an extension of human abilities, for example for analysis, gaming, as part of a service-related inquiry, or as learning partner (e.g. [53]), (b) chatbots supporting human collaboration, for example by taking notes, documenting, or task management (e.g. [104]), and (c) chatbots collaborating with other services for example in multi-agent models, networks of chatbots, or external web services (e.g. [108]).

Chatbots may be integrated into collaborative processes forming what Grudin and Jacques [45] refer to as *humbots*, that is, human-chatbot teams which handle challenging service queries better than chatbots alone and more efficiently than humans alone. The concept of humbots assumes a tiered approach to service provision where the chatbots constitute an initial service contact point, and customers are escalated to human helpers only if the chatbot is unable to help. Such human–chatbot teams draw on the concept of human-in-the-loop [24] from the human factors literature, sensitizing system managers to the need for a collaborative setup allowing sufficient situation awareness to the human part of the team to provide quality takeover if need be. In health-care context, human-in-the-loop concepts for conversational agents supporting hospital nurse teams has proved beneficial [13]. Likewise, the notion of escalation in customer service chatbots is a practical application of the human-in-the-loop concept for robust application of chatbots in consumer service provision [83].

#### Research challenges

There is an essential challenge in studying and designing chatbots for collaboration due to the multifaceted character of such interaction, and the range of potential theoretical perspectives to apply. For example, collaboration may be framed line with game theory—where an agent can be either a collaborator or an opponent [56]—or follow joint-intention theory where an agent is always aimed to work together with the user [55, 68] or to establish a partnership [31]. When setting the concept of collaboration within social settings, the agent may be considered a mediator of human actors rather than an established actor within the described social structure (e.g. [104]). Or collaboration is addressed as merely a technical feature when the agent is collaborating with other artificial agents and external web services (e.g., [108]).

While a range of chatbots for collaboration have been developed, there is relatively scarce research on the characteristics of collaboration with chatbots. That is, we lack models or theories helping us to conceptualize collaboration involving intelligent conversational agents. While this problem of human–machine collaboration is addressed in more generic terms, for example in actor-network theory [70], there is a lack of models to characterize conversational collaboration involving agents. Related to the challenge of conceptualizing chatbot collaboration, there is a need for research on the different roles chatbots and humans should take in the human–chatbot collaboration, and what the implications of these may be. Should for example, the relation be based on assistance or mutual collaboration? Should chatbot participation be reactive or active? Should the chatbot be submissive or take charge? And what would the implications of these choices be?

#### Future research directions

Drawing on the above state of the art and research challenges, the following research directions are found to be particularly promising.

- *Modelling human–chatbot collaboration.* Research is needed to conceptualize and model different forms of human–chatbot collaboration, the roles the collaborative partners may take, and the potential implications these forms and roles may have in the short and long term. Addressing this complex concept within interactions with a novel technology like chatbots may benefit from inductive approaches. Future research may build theory inspired by knowledge on collaboration with humans and other artificial agents than chatbots—such as social robots and embodied virtual agents. Accordingly, the concept of collaboration could be conceptualized in line with chatbots' unique embodiment features, paying particular attention to the possible roles of chatbots in collaboration and identifying properties which express these.
- *Empirical investigations of human–chatbot collaboration.* When robust concepts for human–chatbot collaboration are established, a range of exciting empirical research is foreseen—for example involving experimental studies and case studies. As part of such research, it will be valuable to investigate incentive structures in collaboration, instruments for measuring human–chatbot collaboration, task-specific differences in outcomes, and levels of participant engagement and activity across and within tasks. These may also be included as mediators, moderators and covariates in complex behavioural models studying other concepts as outcomes (e.g., customer satisfaction, user experience, or technology adaptation). Thus, collaboration with chatbots could be situated not solely as an outcome or a predictor, but also as an adaptive behaviour that has a substantial role in a variety of settings and applications.

### Democratizing chatbots–chatbots for all

The topic of democratizing chatbots concerns how chatbots may be developed, designed, and deployed to improve availability and accessibility to information and services. Furthermore, how chatbots may be beneficial in bridging digital divides across various user populations. By nature, democratizing chatbots is a topic of interest to the human–computer Interaction community, but not limited to it. Any discussion around democratizing chatbots has at least some overlap with larger debates concerning the ethics of artificial intelligence—in particular for issues pertaining to fairness, non-discrimination, and justice [47].

#### State of the art

By allowing simple natural language dialogues, chatbots are potentially a low-threshold means to access information and services and may as such serve to bridge digital divides and strengthen inclusion [14]. Chatbots have been suggested as accessible interactive systems for visually impaired in need of an easily navigable user interface [7], as conversational support for users with special needs [19], and to support youth to engage in societal issues [110]. Chatbots may improve access to health care services (e.g. [105]) and support health-promoting behavior change (e.g. [85]), and supplement educational programs [53].

Also relevant for the democratization of chatbots is also the relative lowering of thresholds that chatbots may introduce to interactive systems development and design. A number of current chatbot platforms are marketed under the promise of supporting chatbot design without need for coding skills [26]. Likewise, to involve domain experts in dialogue design, platforms may include dashboards for low-code updates of chatbot content and interaction design [66] or take up low-code approaches [89]. However, to our knowledge, there is a lack of research on the usability, accessibility, and effectiveness of such platforms.

However, some studies highlighted critical aspects in using chatbots since they may sustain and even strengthen existing biases in society. For example, a gender bias has been identified in chatbot design [33], and voice-based conversational agents have been shown to more easily interpret particular English dialects potentially reducing their utility for users of specific areas [51], and also to be difficult to use for user groups with speech impairments [19]. Although many major companies, research institutions, and public sector organizations have issued ethical artificial intelligence guidelines, recent work [58] has discovered substantial divergence in how these are written and interpreted, highlighting the complexity of designing guidelines for systems with complex social impact. In this way, responsibility is placed on designers and developers to cultivate awareness of these issues and how their approaches impact the end-user instead of discussing shared ethical approaches and focusing on agent decision-making.

#### Research challenges

Recent studies suggest that while chatbots may indeed serve as a low-threshold interface to information, services, and societal participation, they may also face challenges regarding bias and inclusion. Besides, there is a lack of more systematic or structured investigations of universal and inclusive design of chatbots. Inclusive and responsible design of chatbots requires an understanding of various linguistic elements of conversation and an awareness of broader social and contextual factors. For example, studies are needed on barriers to onboarding and barriers to the use of chatbots. The aim of using chatbots for strengthening democratization, reducing bias, and facilitating universal design has been included in the vision of *chatbots for social good* [40], which may be a useful scope for addressing this set of challenges.

Furthermore, while available platforms and frameworks are promoted as low-threshold means of chatbot design and development, there is a lack of knowledge regarding how these are actually employed to democratize chatbot development and design. Also, knowledge is needed on what challenges users with limited technology skills meet when trying to use these platforms and frameworks, and how such challenges may be overcome through changes, for example, in design and training of machine learning models.

#### Future research directions

In light of the background and research challenges mentioned above, the following broad directions of future research are identified.

- *Chatbots for social good. *To realize the potential of chatbots as vehicles for bridging digital divides and strengthening accessibility, availability, and affordability of services and information, chatbots for social good may be leveraged as an alternative perspective on chatbot research and design. In this perspective, systematic studies are needed to gain insight into current barriers in chatbot use how these could be employed for social good. In this way, it will be possible to seek to overcome the existing barriers with standardized solutions and follow user-centered design processes focusing on user needs. Finally, research is needed on the normative and ethical implications of the adoption of chatbots in particular contexts, as also outlined in the next section.
- *Inclusive design with and for diverse user groups.* Parallel to the research direction of chatbots for social good, we foresee research and development continuing the work towards making the underlying platforms and frameworks for chatbot design and development more easily applicable also for users without strong technical skills. Here, we foresee studies of current opportunities and challenges faced by chatbot creators, followed by development and design stages, aiming to follow up or mitigate these. Removing the need for complex configuration and simplifying or eliminating coding is probably the easiest way to serve the needs of the small business and research groups—but also the needs of large enterprises that may have domain experts creating chatbots. Furthermore, developing platforms that facilitate the implementation of chatbots and recommend using best practices during the design process will surely raise the quality level of the final products.

### Ethics and privacy in chatbots

The final research topic concerns ethical and privacy implications of chatbots. Specifically, how to reflect ethical and privacy concerns in the design of chatbots, recognising the implications that different chatbot use cases and design choices may have for users’ trust in chatbots, and how we may identify and address unethical chatbot use.

#### State of the art

AI has recently been the objective of substantial interest from policy-making and regulatory bodies, as well as in discussions and reflections on ethics, privacy management and trust [21]. This concern for ethics in AI is motivated by its disruptive character and potential for changes in the job market and misuse by malevolent actors, as well as issues pertaining to accountability and bias [58]. Ethical concerns arising from the design and deployment of AI technology have motivated a number of initiatives [47]—such as the Ethical Guidelines for Trustworthy AI by the European Commission expert group on AI, and Microsoft’s FATE: Fairness, Accountability, Transparency, and Ethics in AI—addressing issues including mitigating bias and discrimination in AI systems and fairness in the use of AI systems [81]. Chatbots are a prominent AI-based technology, and as such in principle addressed by the broader concern for ethics and privacy in technology research in general and in AI-based technology in particular. Nevertheless, as noted in a review of the chatbot literature, there has been an initial lack of ethical discussion in chatbot research [102]—though noteworthy exceptions to this exist, such as the exploration of ethical and social considerations for conversational AI by Ruane et al. [91]. The ethical discussion in chatbot research may, however, be gaining traction motivated, for example, by Bender et al.’s [9] critical overview of ethical risks pertaining to large language models.

The interest and discussion concerning ethics and privacy in AI have been particularly impactful in Europe, where the General Data Protection Regulation (GDPR) is now used to govern privacy in technology-based systems and services. Furthermore, based on the advice of a high-level expert group on AI, a European set of ethics guidelines for trustworthy AI has been presented [30]. According to these guidelines, it is of paramount importance for trustworthy AI to be aligned with (a) legal regulations and (b) ethical principles and values, and also (c) be robust from a technical perspective given its particular social context. From these principles, the European Commission expert group has identified seven key requirements for ethical AI applications, including human agency and oversight, privacy and data governance, and diversity, non-discrimination, and fairness. Finally, a proposed European set of regulations for AI, the AI Act, will help strengthen aspects of ethical concern in AI systems, including legal requirements for human oversight, accuracy, robustness, and security. Of particular relevance for chatbots is the proposed requirement for transparency which will make it an obligation for service providers to ensure users are aware when are interacting with machine agents and not human operators [94].

#### Research challenges

Ethical and privacy challenges permeate the field of chatbot research, but specifically where the context is sensitive or high-stakes or the users are marginalised or vulnerable; for example, in designing chatbots for health and education, or in designing chatbots to support asylum seekers or children. There is a large and growing body of ethical and privacy knowledge to draw on, and an emerging set of guidelines and regulations on ethics and privacy for digital systems in general, and AI-based systems in particular. Nevertheless, we lack research and theorising around ethics and privacy specifically for conversational user interfaces. This is problematic, as the conversational character of chatbots may conceivably introduce a range of specific ethical problems, for example the ethical implications of human-like and socially present chatbot interaction, issues of consent, the privacy implications of third-party interactions and the implications of emotional effect on children and vulnerable users. Research is needed to better understand and address these, and other, emergent problems of ethics and privacy.

#### Future research directions

Drawing on the above, we accentuate the following two directions for future research—though other directions could be possible and maybe equally relevant.

- *Understanding chatbot ethics and privacy.* Future research should facilitate reflections on ethical implication of chatbots, for example through identification of ethical and privacy issues in chatbot design and implementation—including design intentions, practical mitigation of known issues and exploration of unforeseen implications. These could be domain-specific issues, such as ethical implications for research and education, media or marketing and commerce, but these could also be general issues such as how interaction with chatbots may motivate oversharing in users, helping spread misinformation and hate speech, or induce potential negative consequences as a result of over-humanizing chatbots.
- *Ethics by design.* In parallel with work on chatbot ethics, there will be a need for research on the pragmatic and material issues of how to honour ethical guidelines and principles in the design of chatbot technology and applications. With reference to the principle of *privacy by design*, we refer to this as *ethics by design*—where privacy is subsumed as one of several aspects to consider as part of an ethics discussion and subsequent design challenge. Important challenges may include research on how to avoid biases in chatbots, how to avoid chatbot discrimination and redlining, and how to mitigate the ethical issues introduced by the black-box approach to machine learning underpinning aspects of chatbot functioning, as well as to avoid misuse and weaponization of chatbot technology. A useful starting point for an exploration of ethics by design, could be to refine the generic European expert group requirements for ethical AI [30] to the context of conversational AI.

## Discussion

Drawing on the involvement of chatbot researchers and practitioners in the CONVERSATIONS workshops, we propose a set of future directions for chatbot research. The directions are motivated by the current state of the art and identified research challenges and structured within six overarching topics. In the following, we discuss the implementation of the future directions, our perspectives on chatbot application areas, and how to continue the discussion and reflection started in this paper.

### Implementing the future directions

Two of the identified research directions concern studies of users and implications, as well as how to design for desirable chatbot use. As chatbots become more pervasive in the coming years, and communication with non-human agents increasingly become part of our daily routines, it becomes even more pressing to expand our knowledge on the antecedents, contents and consequences of human–machine communication. In doing so, this stream of research needs to explore the cognitive, affective and behavioural dimensions of engagement with these agents, the extent to which there are systematic differences between individuals, groups or contexts of use, and the individual, group and societal implications of this phenomenon. Moreover, as the field progresses, there is a growing need to consolidate the existing knowledge, updating and extending overarching theoretical frameworks and models. Work within a wide variety of disciplines can serve as an inspiration in that regard, such as the studies of Sundar [100] on the psychology of human–agent interaction and Guzman and Lewis [46] on human–machine communication.

This evolution in our understanding of conversational user experiences should be accompanied with the proper support from platforms and frameworks. We can see the support as increasingly creating abstractions that would facilitate the design, testing, integration and development of chatbots, as it has historically happened with other software artefacts. Current efforts are already moving in that direction, providing development resources that promise anyone with enough motivation, regardless of their background, to deliver human-like interactive experiences. While this has the potential to bring substantial value to societies, empowering communities to develop their own solutions, it can also bring unintended consequences, as we cannot expect users of these platforms to have knowledge about complexities of modelling proper Human-AI experiences [116]. On the other hand, abstractions can also hide underlying information about machine learning models, AI decision-making, as well as latent bias in the training data (e.g., [101]) that can translate into social biases (e.g., [120]).

Human–chatbot collaboration is foreseen as an increasingly important aspect of chatbot research and applications. We hold that such collaboration will benefit from being implemented while reflecting on human collaboration, and in line with relevant empirical evidence of chatbot research—in line with reflections by Grudin and Jacques [45]. Considering the meaningful value of collaboration for decision making and productivity in professional and organizational settings, tasks assigned to chatbots in these collaborative interactions can vary in complexity and involvement. Such tasks can be as simple as providing individual notifications, or as complicated as communicating processed and analysed data to different stakeholders. Using chatbots for automating these tasks should enrich group productivity and quality of work, promoting mutual understanding and diversity of opinions. Research supporting such automation could benefit from seeing this as a service design challenge, where the chatbot is seen as one of multiple agents and user interfaces [14]. On a societal level, collaborative networks of humans and chatbots may require new online safe spaces, with chatbots demonstrating higher levels of involvement. These can moderate social interactions, facilitating engagement, inclusivity and understanding within the parties involved. This is in stark contrast to the current challenge of software agents or bots in social networks, as for example seen in Twitter bots utilizing COVID-19 content to spread political conspiracies [34], and the general trend of deploying bots in large scale for political interference and influence [44].

Chatbots will both raise critical ethical challenges and hold implications for democratization of technology, and implementing research addressing these directions is important. Chatbots permit users to interact through natural language, and consequently are a potential low threshold means to access information and services and promote inclusion. However, due to technological limitations and design choices, they can be the means to perpetuate and even reinforce existing biases in society, exclude or discriminate against some user groups (e.g. [33, 51]) and over-represent or enshrine specific values. Future research will need to investigate and demonstrate democratization of chatbots in practice, where conversational technology is to be made easily and widely accessible for various businesses and user groups across the globe so that more people can benefit from conversational interaction. Moreover, as part of chatbot democratization, it will be important to make their development process more accessible as well, without requiring chatbot developers to have in-depth software engineering knowledge—as exemplified in using visual programming approaches such as Blockly to chatbot development [89]. In this way, chatbots can be created by experts in the domain where they will be used. This aspect is fundamental since chatbots are not conventional technologies, but they are developing as agents operating in social contexts.

Taking a broader ethical perspective, key questions when implementing future research on chatbots may include: What are the ethical implications of chatbots imitating human beings? Whose (and which) values should guide design practice within a global marketplace? What are the ethical implications of replacing humans with chatbots as a means of support for purposes such as commerce, therapy, or social interaction? How to facilitate chatbot support in decision making without risking or compromising agreed ethical principles? Ethical reflections and discussion on chatbots and chatbot applications are already emerging (e.g. [65, 91]). We anticipate that advances in the democratization of chatbots will increasingly inspire ethical discourse that ties in with higher-level discussions about chatbot applications.

### Perspectives on chatbot application areas

The identified research topics and corresponding future research directions may guide research so as to contribute to the fundamental understanding of the chatbot technology and the corresponding user interaction and engagement. However, to generate added value in specific application areas—such as customer service, health, education, office work, and home applications—further reflections about the respective use cases are needed. In particular, researchers need to analyse how chatbots may be leveraged and taken up in different application areas, how knowledge and research may be transferable across different application areas, and whether distinct research agenda should be established.

Many aspects of the outlined on our future research agenda are valid for any application area. For example, results concerning chatbot communication, user experience, design, and technology are the basis for applying chatbots in specific application areas. However, further analysis efforts are needed to understand the characteristics of each application area in more detail. For instance, requirements in the health sector concerning privacy aspects, ethics, and trust may be significantly more demanding than similar requirements in other sectors as they might have severe impacts on the users and concern highly sensitive personal information. In business contexts such as corporate customer support scenarios, the potential impacts may be less severe, but specific corporate regulations and norms need to be considered. In contrast to that, the use of chatbots in personal settings, e.g. the use of chatbots for social relationship, is often mainly driven by motivations for engagement and meaning-making. In contrast to health and business contexts, personal benefits are often not measured in monetary terms, but the main focus of personal usage is the improvement of daily life or wellbeing.

Regardless of differences among the diverse application areas in application-oriented research, many research studies exist in specific domains that could possibly be transferred to others. For instance, studies focusing on information provision in business contexts can most likely be applied in the health sectors as well, e.g., provision of product information will likely be similar to explaining healthy nutrition. However, to enable a transfer of research results across application areas, commonalities and differences of the involved application areas need to be identified and assessed. If the main characteristics of both are similar, transfer of the research results seems viable. Based on such an analysis and comparison, a generalization of the research across application areas seems possible. This procedure for future research on chatbot application areas could lead to a substantial increase in the body of knowledge as many research results from existing pilot studies and prototypes for specific application areas exist and may be reused as the basis for transfer and generalization (i.e., general design guidelines) to further application areas.

### Continuing the discussion and collaboration

The presented challenges may serve as a step in the direction of contributing to the body of knowledge about chatbot usage and challenges, the frameworks and platforms underpinning chatbot applications, as well as needed future work on the broader implications of chatbots to work and society.

The proposed future research directions are intended as a response to the current lack of coherence in the emerging field of chatbot research, which may in part be observed by the broad range of journals and conferences in which findings from chatbot research are presented, and also the lack of commonly agreed key constructs, models, and measurement instruments. While this may be expected in an emerging research area, future research will benefit from a greater degree of coherence and cohesiveness in the field.

Nevertheless, there may be topics that have been omitted in the process leading up to our proposition, and relevant state-of-the-art and current research challenges may have been left out. Furthermore, as the field evolves, it is necessary to update the set of topics and research directions regularly. In consequence, continued interdisciplinary discussion and collaboration are needed to validate and refine the proposed set of future research directions.

One limitation deserving particular mention concerns the context of this discussion. The findings are based on discussions at the CONVERSATIONS workshop and mainly involve researchers from European organizations. While we assume the proposed directions hold broad international relevance and interest, it may be fruitful to test this assumption through discussion in the field—a discussion which we hope this paper will spur.

In further discussion and collaboration on chatbot research directions, care should be taken to involve the broadest possible set of interests and perspectives. For example, it will be beneficial to involve both researchers and practitioners, as well as the emerging and established set of research communities with an interest in conversational computer systems, to make sure that different enabling technologies and knowledge resources needed in future development and design of chatbots are represented. While research on conversational systems and user interfaces has been conducted for decades, chatbot research and design are still in its relative infancy. A consolidation of the field is needed, and we hope the proposed research agenda, with its directions for future research, may serve as a step towards such consolidation.

## Footnotes

1 DialogFlow, https://cloud.google.com/dialogflow.

2 Microsoft Bot Framework, https://dev.botframework.com/.

3 Pandorabots, https://home.pandorabots.com/.

4 Rasa, https://rasa.com/.

5 Mycroft, https://mycroft.ai/.

6 CUI 2021—Conversational User Interfaces, https://www.conversationaluserinterfaces.org/2021/.

7 CONVERSATIONS 2021—international workshop on chatbot research, https://conversations2021.wordpress.com/.

8 CAIR 2020—Conversational Approaches to Information Retrieval, https://sites.google.com/view/cair-ws/cair-2020.

9 SIGDIAL—Special interest Group on Discourse and Dialogue, https://www.sigdial.org/.

10 IVA 2021—21st ACM International Conference on Intelligent Virtual Agents, https://sites.google.com/view/iva2021/.

11 IWSDS 2021—12th International Workshop on Spoken Dialogue Systems Technology, https://www.iwsds.tech/.

12 INTERSPEECH 2021—The 21st Annual Conference of the International Speech Communication Association, https://interspeech2021.org.

13 Google Conversation Design, https://designguidelines.withgoogle.com/conversation/.

14 Alexa Design Guide, https://developer.amazon.com/en-US/docs/alexa/alexa-design/get-started.html.
